# Roles of microRNA-124 in traumatic brain injury: a comprehensive review

**DOI:** 10.3389/fncel.2023.1298508

**Published:** 2023-11-28

**Authors:** Panxing Wu, Bao He, Xiaoliang Li, Hongwei Zhang

**Affiliations:** ^1^Department of Neurosurgery, Taizhou Central Hospital (Taizhou University Hospital), Taizhou, Zhejiang, China; ^2^Department of Neurosurgery, The First People’s hospital of Kunshan, Affiliated Kunshan Hospital of Jiangsu University, Suzhou, Jiangsu, China; ^3^Suzhou Key Laboratory of Neuro-Oncology and Nano-Bionics, Suzhou, Jiangsu, China; ^4^Department of Emergency Medicine, Taizhou Central Hospital (Taizhou University Hospital), Taizhou, China

**Keywords:** traumatic brain injury, target, function, mechanism, microRNA-124

## Abstract

Traumatic brain injury (TBI) is a prominent global cause of mortality due to the limited availability of effective prevention and treatment strategies for this disorder. An effective molecular biomarker may contribute to determining the prognosis and promoting the therapeutic efficiency of TBI. MicroRNA-124 (miR-124) is most abundantly expressed in the brain and exerts different biological effects in a variety of diseases by regulating pathological processes of apoptosis and proliferation. Recently, increasing evidence has demonstrated the association between miR-124 and TBI, but there is still a lack of relevant literature to summarize the current evidence on this topic. Based on this review, we found that miR-124 was involved as a regulatory factor in cell apoptosis and proliferation, and was also strongly related with the pathophysiological development of TBI. MiR-124 played an essential role in TBI by interacting with multiple biomolecules and signaling pathways, such as JNK, VAMP-3, Rela/ApoE, PDE4B/mTOR, MDK/TLR4/NF-κB, DAPK1/NR2B, JAK/STAT3, PI3K/AKT, Ras/MEK/Erk. The potential benefits of upregulating miR-124 in facilitating TBI recovery have been identified. The advancement of miRNA nanocarrier system technology presents an opportunity for miR-124 to emerge as a novel therapeutic target for TBI. However, the specific mechanisms underlying the role of miR-124 in TBI necessitate further investigation. Additionally, comprehensive large-scale studies are required to evaluate the clinical significance of miR-124 as a therapeutic target for TBI.

## Introduction

Traumatic brain injury (TBI) is conventionally characterized as the disturbance of regular cellular function within the brain resulting from direct, rotational, and shear forces, such as falls, blows, or blasts. TBI encompasses distinct classifications, namely closed-head TBI and open TBI (also referred to as penetrating TBI). Additionally, TBI is further stratified into mild, moderate, and severe categories based on the extent of the condition. The principal clinical manifestations of TBI include coma, headache, seizures, and alterations in behavior (Godoy and Rabinstein, 2022). The pathological progression of TBI comprises two stages: primary injury and secondary injury. TBI not only typically produces an immediate tissue injury, but also induces long-term neuropathological changes, including disruption of blood–brain barrier permeability, oxidative stress, and cognitive deficits (Zhu et al., 2021). In addition, there might be a positive association between TBI and long-term neurodegenerative disorders, including Parkinson’s disease and Alzheimer’s disease (VanItallie, 2019).

Despite advances in treatment of TBI in recent decades, patient outcomes remain poor. TBI is reported to be a leading cause of death worldwide. Along with industrialized development, the incidence of TBI is increasing. TBI will not only significantly reduce quality of life, but also impose an economic burden worldwide. The global number of TBI is estimated at 69 million per year (Bourgeois-Tardif et al., 2021). During the past three decades, both incidence and prevalence rates have elevated globally (GBD 2016 Traumatic Brain Injury and Spinal Cord Injury Collaborators, 2019; Dams-O’Connor et al., 2023). It is common for patients with moderate-to-severe TBI to suffer lasting functional impairment. Treating TBI and associated comorbidities is estimated to cost 406 billion annually worldwide (Maas et al., 2017). Individuals with objective similar injuries can experience highly disparate outcomes, ranging from full recovery to substantial disability or death (Dams-O’Connor et al., 2023). To date, no known treatments are currently available to delay or prevent the progression of post-TBI pathologies. Therefore, further research on the potential mechanism of TBI is urgently needed to explore new therapeutic targets and improve the prognosis of TBI. Now, increasing studies prove that microRNAs (miRNAs) may play an essential role in the progression of post-TBI pathologies.

MicroRNAs are short and non-coding RNA molecules 19–25 nucleotides in size that promote the degradation of mRNA by binding the 3′ untranslated region of the target gene mRNA, thus regulating post-transcriptional silencing of target genes (Hill and Tran, 2021). A single miRNA can regulate hundreds of mRNAs and influence the expression of many genes. Recently, miRNAs have been shown to participate in the pathogenesis of many diseases, including TBI, spinal cord injury, and tendon injury (Peng et al., 2020; Herrold et al., 2021; Liu et al., 2021; Garcia et al., 2022a). Meanwhile, miRNAs have been proposed as suitable targets for the treatment of many diseases, including TBI (Xiao et al., 2019; Yin et al., 2020). Yin et al. (2020) reported that the expression of miR-21-5p was increased in neurons and microglia after TBI. Furthermore, miR-21-5p promoted the apoptosis of neuron cells by inducing microglia polarization and aggravating the release of neuroinflammation factors (Garcia et al., 2022a). Garcia et al. (2022a) showed that miR-21 and its target gene PPARα might be the promising biomarkers for Alzheimer’s disease. Interestingly, miR-21 mimic treatment was found to be responsible for the neuroprotection of post-stroke brain damage (Lopez et al., 2022). Xiao et al. (2019) found that prostaglandin-endoperoxide synthase-2 (Ptgs2), also known as cyclooxygenase-2, was significantly up-regulated whereas miR-212-5p was decreased in the TBI group compared to the sham group. Further study showed that overexpression of miR-212-5p significantly improved learning and spatial memory in TBI mice by attenuating ferroptosis through the inhibition of Ptgs2 (Xiao et al., 2019). A recent study showed that miR-124 was also involved in the progression of TBI (Schindler et al., 2020). miR-124 is the most abundant of miRNAs in the brain (25–48% of all brain miRNAs) (Lagos-Quintana et al., 2002). Also, miR-124 is one of the most well-studied miRNAs in the nervous and immune systems (Han et al., 2019). Additionally, it is vital for neuronal development and immune responses (Li et al., 2021; Sanuki and Yamamura, 2021). A previous study developed by Liu et al. (2015) demonstrated that the serum miR-124 level decreased significantly within 24 h of acute ischemic stroke. The authors also found that serum miR-124 within 24 h was negatively associated with a high level of C-reactive protein (an indicator of inflammation) (Liu et al., 2015). This study indicates that miR-124 expression might be correlated to development of acute neuropathy. In recent years, the role of miR-124 in TBI has received increasing attention among researchers. The latest relevant study conducted by Zhuang et al. (2023) showed that miR-124 was involved in the improvement of neurological damage in TBI exerted by bone marrow stromal cells-derived exosomes via the p38 MAPK/GLT-1 axis.

After a comprehensive search, we identified a certain number of studies that reported on the essential roles of miR-124 in TBI, either clinical trials or experimental studies. Currently, however, no review is available for summarizing the evidence of specific roles of miR-124 in TBI. Since TBI is amongst one of the most life-threatening illnesses worldwide, a better knowledge of the biological function and clinical significance of miR-124 in TBI is of great clinical importance. Systematically searching was performed in the four common electronic databases, including the MEDLINE, Embase databases, Cochrane Library databases, and the PsychINFO. The timeframe spanned from the inception of these databases to March 1, 2023. The search terms used in the MEDLINE were miR-124 (microRNA-124) and traumatic brain injury (TBI). We selected any studies that reported the roles of miR-124 in TBI, either clinical trials or experimental studies. In the MEDLINE database, the selection procedure for screening the potentially included studies depended on the inclusion criteria. In total, 74 studies were found in the four databases during the initial search. Forty-one articles were excluded after removing duplicates. Twenty-five studies were assessed for eligibility. After removing those studies that failed to meet the inclusion criteria, article correction, and review articles, sixteen studies were finally included. Supplementary Figure 1 shows the flow chart of the study selection.

In this review, we mainly summarize the current knowledge about miR-124 in the progress of TBI. Table 1 lists the characteristics of the main findings of the sixteen included studies.

**TABLE 1:** Characteristics of the sixteen included studies.

References	Research subject	Brain injury type	Measure method	Expression of miR-124	Main findings
Ge et al., 2020	Mice and BV2 microglial cell	rmTBI	qRT-PCR	Down-regulation	miR-124-3p alleviated neurodegeneration and improve the cognitive outcome by inhibiting Rela expression and promoting ApoE expression after rmTBI.
Huang et al., 2018	Mice and BV2 microglial cell	rTBI	ELISA	Down-regulation	miR-124-3p improved the neurologic outcome by suppressing neuroinflammation in mice with rTBI through the inhibition of PDE4B/mTOR pathway.
Yang et al., 2019	Rat and BV2 microglial cell	TBI	qRT-PCR	Down-regulation	miR-124 improved function recovery after TBI by promoting M2 polarization of microglia through the inhibition of TLR4 pathway.
Zhao et al., 2022	BMVECs	TBI	qRT-PCR	Down-regulation	MiR-124-3p promoted autophagy by inhibiting mTOR signaling, thereby protecting cells against TBI-induced damage.
Chen et al., 2019	Rat and microglial cell	TBI	qRT-PCR	Down-regulation	MiR-124 ameliorated surgical stress-induced microglial activation by preventing proinflammatory cytokine release via inhibiting VAMP3 expression in the hypothalamus and hippocampus.
Li X. L. et al., 2022	Rat and microglial cell	TBI	qRT-PCR	Down-regulation	miR-124-3p inhibited the TBI-associated inflammatory by suppressing MDK and TLR4/NF-κB expression.
Shi et al., 2022	Patients and mice	TBI	qRT-PCR	Down-regulation	miR-124-3p improved memory and motor behavioral tests by inhibiting DAPK1 expression and reducing NR2B phosphorylation in TBI mice.
Vuokila et al., 2018	Rat	TBI	Droplet digital PCR, situ hybridization, and hematoxylin and eosin (H&E) staining	Down-regulation	Inhibition of the miR-124-3p promoted injury neurogenesis and the inflammatory response by targeting STAT3 and Plp2 after TBI.
Vuokila et al., 2020b	Patients and rat	TBI	RT-PCR and situ hybridization	Down-regulation	Downregulation of miR-124-3p in the perilesional cortex led to post-injury neurodegeneration and inflammation.
O’Connell et al., 2020	Patients	TBI	qPCR	Up-regulation	The expression level of miR-124-3p was elevated in patients with TBI.
Vuokila et al., 2020a	Rat	TBI	RT-PCR and Droplet digital PCR	Up-regulation	The extent of TBI was associated with the elevated plasma miR-124-3p level. This increasement was correlated linearly to the extent of the chronic loss of cortical tissue.
Li et al., 2019	BV2 microglial cell	rTBI	qRT-PCR	Down-regulation; after treatment: upregulation	Upregulation of miR-124-3p following TBI inhibited neuronal autophagy and protected against nerve injury through their transfer into neurons.
Yip et al., 2019	Mice and BV2 microglial cell	TBI	Fluorescence *In situ* hybridisation (FISH) and Immunohistochemistry	Down-regulation; after treatment: upregulation	DHA induced neuroprotection in contusion injury by increasing the expression of miR-124.
Kang et al., 2022	Rat	TBI	qRT-PCR	Down-regulation; after treatment: upregulation	The miR-124-3p antagomir improved the motor function of TBI rats by promoting the PI3K/AKT and Ras signaling pathways.
Johnson et al., 2017	Rat	TBI	qRT-PCR	Down-regulation	The miR-124a expression of brain tissue increased at 1 day post-injury and was inversely associated with inflammatory proteins, IL-6 and IL-1beta.

rmTBI, repetitive mild traumatic brain injury; rTBI, repetitive traumatic brain injury; PDE4B, phosphodiesterase-4 subtype B; mTOR, mammalian target of rapamycin; TBI, traumatic brain injury; VAMP3, vesicle-associated membrane protein 3; MDK, midkine; TLR4, toll-like receptor 4; NF-κB, nuclear factor-kappaB; DAPK1, death-associated protein kinase 1; NR2B, N-methyl-d-aspartate receptor subunit 2B; RGCs, retinal ganglion cells; STAT3, signal transducer and activator of transcription 3; Plp2, proteolipid protein 2; DHA, docosahexaenoic acid; PI3K, phosphatidylinositol 3-kinase; GAS5, growth arrest-specific transcript 5; JAK, Janus kinase; JNK, c-Jun N-terminal kinase.

### Known roles of miR-124

In 2002, miR-124 was first identified in mice (Dash et al., 2020). miR-124 is highly conserved and widely expressed in both humans and murine (Kozuka et al., 2019). Up to date, three miR-124 isoforms miR-124-1, miR-124-2, and miR-124-3 have been identified based on different chromosome locations (Zeng et al., 2021). Promoters at the above chromosome locations all contain CpG islands. CpG methylation plays an important role in promoting the silencing effect of the miR-124 encoding gene, causing abnormal expression of miR-124 and inactivating the miR-124 target mRNA (Zeng et al., 2021). These regulatory networks exert different biological effects on various human diseases. The mature miR-124 generation process is relatively complicated. First, the miR-124-encoded gene is transcribed into primary miR-124 (pri-miR-124) through RNA polymerase II (Karam et al., 2022). Second, pri-miR-124 can be recognized by the Drosha-Database of Gene Co-Regulation 8 (DGCR8) complex, and this complex cuts it into 70 nucleotides (nt) long precursor of miR-124 (pre-miR-124) (Wang et al., 2017). Subsequently, pre-miR-124 is further processed into 21 nt duplex miRNA (Guo et al., 2022). Finally, one strand of the duplex miRNA becomes a mature miRNA (also known as miR-124-3p or 5p), and the other strand is degraded by helicase (Yang et al., 2021). According to “miRbase,”^1^ an online tool for microRNA research, the previous ID for miR-124-3p is miR-124 and miR-124a, while miR-124-5p is a subsequent miRNA belonging to the family of miR-124. Both miR-124-3p (miR-124) and miR-124-5p are mature miRNAs and their biological functions might be different. But all the included studies reported rather miR-124 (miR-124-3p) than miR-124-5p. Mature miR-124 exerts regulatory control at the posttranscriptional level by degrading or repressing target gene translation through targeting complementary mRNA sequences (Zhang et al., 2021).

Over the past decade, miR-124 has become a hot research spot. Numerous studies showed that miR-124 played an important role in a variety of biological processes, including apoptosis (Xue et al., 2023), proliferation (Song et al., 2023), and migration (Ghafouri-Fard et al., 2021), and was significantly differentially expressed in various tumors (Geng et al., 2021), inflammatory diseases (Tazi et al., 2021), and neurological diseases (Sanuki and Yamamura, 2021; Zhao et al., 2021). It was reported that miR-124 suppressed tumor progression by regulating different target genes. For example, Liu T. et al. (2020) showed that AKT2, playing a pro-oncogenic role in many human cancers, was a miR-124 downstream target gene and that overexpression of miR-124 in non-small cell lung cancer (NSCLC) led to downregulation of AKT2 and played a tumor suppressor role. In addition, miR-124 was reported to have a significant neuroprotective function in neurological diseases, including Alzheimer’s disease (AD) (Ouyang et al., 2022). A previous study indicated that miR-124 and its targeted gene BACE1 collectively contributed to the pathogenesis of AD (An et al., 2017). Garcia et al. (2021) revealed that miR-124 might be crucial for proper neuronal function in AD models. Kang et al. (2017) found that miR-124-3p mimics significantly attenuated neuronal cell apoptosis by inhibiting abnormal hyperphosphorylation of Tau through the Caveolin-1-PI3K/Akt/GSK3β pathway in AD. In line with this, another study conducted by Gu et al. (2018) demonstrated that miR-124 promoted neurite development by regulating the HDAC5-MEF2C-M6a pathway in primary neurons. Neuronal cell apoptosis is the major cause of neurological deficits (Yang and Liao, 2022). A recent study showed that the extent of TBI was proportional to the elevation of the plasma miR-124-3p (Vuokila et al., 2020b). Therefore, miR-124 may exert an aggravating or protective effect on the progress of TBI by regulating different target genes or signaling pathways.

### The roles of miR-124 in the progress of TBI

#### miR-124-3p alleviates neurodegeneration by inhibiting the deposition of β-amyloid by targeting the Rela/ApoE signaling pathway

A history of TBI has been found to increase the risk of AD due to the neurodegeneration caused by neuronal death (Graham et al., 2022). The heightened risk of AD in TBI is mainly due to the overproduction of β-amyloid (Abu et al., 2018). It was reported that β-amyloid could be regulated by Polymorphisms in the apolipoprotein E (ApoE), encoding apolipoprotein E, a 33–37 kDa glycoprotein (Abu et al., 2018). The ApoE is produced by hepatocytes, macrophages, and adipocytes and influences cellular cholesterol content and lipoprotein metabolism (Abu et al., 2018). Abnormal expression of ApoE is closely associated with TBI. Yu et al. (2021) reported that ApoE ablation before TBI in mice significantly attenuated the development of the spines in the newborn neurons. Decreased levels of miR-124 were found to cause a decline in neurite length and number of spines, while miR-124 inhibitor resulted in the loss of dendritic spines (Garcia et al., 2021). Further studies suggested that ApoE was essential for injury-induced neurogenesis following TBI (Yu et al., 2021). Rela had been found to be an inhibitory transcription factor of ApoE and worsened AD symptoms by promoting the expression of β-amyloid (Xie et al., 2020). A study conducted by Luo et al. (2020) demonstrated that low expression of miR-124-3p promoted apoptosis and ROS production by activating the STAT3/Rela signaling pathway in colonic cells. In addition, the inhibition of miR-124 significantly promoted the production of amyloid-β in the hippocampus of cerebral hypoperfusion rat models (Zhang et al., 2017). However, it is unclear whether miR-124 is involved in TBI by regulating β-amyloid through Rela/ApoE signaling pathway. Recent research studied the change in miR-124-3p expression level in microglial exosomes after repetitive mild traumatic brain injury (rmTBI) and found that the expression level of miR-124-3p gradually increased from 1 to 14 days post-injury (DPI) followed by a slow decline to baseline at 35 DPI (Ge et al., 2020).

The brain-derived neurotrophic factor (BDNF), a member of the neurotrophin family, encourages the differentiation of newborn neurons (Zhu et al., 2019). A previous report demonstrated that serum BDNF and the BDNF-regulatory miR-124 could serve as molecular markers for acute ischemic stroke (Wang et al., 2019). It was suggested that miR-124 attenuated the function of BDNF in activating subventricular zone neural stem cells post-TBI (Kang et al., 2022). Neurogranin, a calmodulin-binding protein, has a neuroprotective effect in some neurological diseases (Xiang et al., 2020). VILIP-1, a neuron-specific calcium sensor protein, is a potential neurodegenerative biomarker and has been found to be elevated in TBI, and early-stage AD patients (Bradley-Whitman et al., 2018; Mavroudis et al., 2021). Ge et al. (2020) reported that repetitive injury reduced the branching of neurites, attenuated neurite outgrowth, increased the expression of VILIP-1, and suppressed the expression of BDNF and neurogranin in neurons, while exosomes with upregulated miR-124-3p (EXO-124) treatment reversed these expression changes. Garcia et al. (2022b) designed a novel SWE cells/secretome (soluble and exosomal) characterized by elevating miR-124, which could translate miR-124 into IFNγ-treated microglia cells and therefore reprogram microglia signature. The authors concluded that miR-124-enriched exosomes might serve as promising therapies in neurodegenerative diseases. Tian et al. (2022) demonstrated that miR-124 and its associated genes played key roles in the action of extracellular vesicles from bone marrow stromal cells to decrease the detrimental effects of stroke on glial cell activation and blood-brain-barrier permeability. These studies suggested that microglial exosomal miR-124-3p inhibited the neurodegeneration induced by repetitive injury. Also, an *in vitro* and *in vivo* study revealed that EXO-124 treatment significantly improved the cognitive outcome after rmTBI (Ge et al., 2020). Further study found that the expression of Rela, amyloid precursor protein (APP), and β-amyloid were increased in injured neurons, while the expression of ApoE decreased in injured neurons (Ge et al., 2020). However, these changes in gene expression could be reversed by EXO-124 treatment (Ge et al., 2020). Moreover, overexpression of Rela significantly blocked the inhibition effect of miR-124-3p on β-amyloid expression (Ge et al., 2020). Similarly, the promotion effect of miR-124-3p on ApoE was also blocked by Rela overexpression (Ge et al., 2020). Additionally, miR-124-3p downregulated the expression of Rela by binding to the 3′UTR sites (Ge et al., 2020). Consequently, microglial exosomal miR-124-3p may mitigate neurodegeneration and enhance cognitive outcomes after rmTBI by suppressing β-amyloid deposition via the Rela/ApoE signaling pathway.

#### miR-124-3p exerts a protective effect in TBI by promoting autophagy through the inhibition of the PDE4B/mTOR signaling pathway

The inflammatory response plays a crucial role in the pathologic development of TBI. High levels of inflammatory cytokines were associated with poor clinical outcomes following the TBI (Malik et al., 2023). Autophagy is a highly conserved, metabolic, and innate immunity process that has impacts on diseases with inflammation, including neurodegeneration, infections, and autoimmunity (Deretic, 2021). Li et al. (2014) reported that autophagy could preserve blood-brain barrier integrity by suppressing vascular endothelial cell inflammation. Mammalian target of rapamycin (mTOR), a serine/threonine kinase, is a well-established suppressor of autophagy and participates in the regulation of various metabolic, survival, and growth-related processes, which is also closely related to the development of Parkinson’s disease (Coleman and Martin, 2022; Wang L. et al., 2022). Tian et al. (2020) demonstrated that calcitonin gene-related peptide (CGRP), a neuropeptide involved in many physiological functions, played an important role in the protection of the injured brain after TBI by inhibiting autophagy through Akt/mTOR signaling pathway. Another study showed that miR-124 influenced Glucocorticoids-induced apoptosis by inhibiting phosphodiesterase 4B (PDE4B) in diffuse large B cell lymphoma cell lines, which was associated with the inhibition of the AKT/mTOR/MCL1 survival pathway (Kim et al., 2015). A recent *in vitro* study demonstrated that the expressions of miR-124-3p, PDE4B, Beclin-1, and proinflammatory cytokines (TNF-a, IL-1b, and IL-6) were increased in the scratch-injury model, while p-mTOR expression decreased (Zhao et al., 2022). Importantly, miR-124-3p overexpression significantly inhibited the expression of PDE4B, p-mTOR and proinflammatory cytokines (TNF-a, IL-1b, and IL-6) and promoted autophagic induction in brain microvascular endothelial cells (Zhao et al., 2022). Furthermore, miR-124-3p overexpression significantly inhibited TBI-induced nerve cell death and this effect was reversed by autophagy inhibitors (Zhao et al., 2022). In addition, PDE4B overexpression blocked the suppressive role of miR-124-3p on mTOR signaling proteins in injured neurons (Huang et al., 2018). These data suggested that miR-124-3p promoted these cells against TBI-induced damage by inducing autophagy through the PDE4B/mTOR signaling pathway. On the contrary, Li et al. (2019) reported that miR-124-3p overexpression exerted a protective effect by suppressing autophagy in scratch-injured neurons. Therefore, further investigation is warranted to elucidate the involvement of autophagy in the progression of TBI.

#### HOXA11-AS aggravates neuroinflammation after TBI by modulating miR-124-3p-mediated MDK-TLR4-NF-κB axis

Long non-coding RNAs (lncRNAs) are involved in various pathophysiological processes after TBI via mediating neuroinflammation and apoptosis. For example, MALAT1 (metastasis-associated lung adenocarcinoma transcript 1) was found to activate after TBI, involving the release of pro-inflammatory mediators and apoptosis of neurons (Patel et al., 2018). Neuroinflammation, a natural reaction after TBI, has been found to exhibit protective effects on the injured brain in a way. However, excessive neuroinflammation might be an important driving reason for delayed hippocampal adult neurogenesis. In patients with TBI, neuroinflammation has been confirmed to contribute to post-traumatic neurodegeneration (van Amerongen et al., 2022). miR-124 was found to be an important biomarker for neuroinflammation in neurological diseases (Yang et al., 2023). In TBI mice, elevated miR-124 in microglial exosomes might remarkably improve the neurologic outcome and suppress neuroinflammation. Meng et al. (2021) reported that lncRNA maternally expressed gene 3 (Meg3) induced microglia inflammation through the miR-7a-5p/Nlrp3 pathway in the pathological process of TBI. Homeobox A11 antisense RNA (HOXA11-AS) is a lncRNA and is associated with inflammatory disease progression. Jin et al. (2018) demonstrated that HOXA11-AS promoted diabetic arteriosclerosis-induced inflammation by activating the PI3K/AKT pathway. Another study performed by Cao et al. (2021) indicated that inhibition of HOXA11-AS significantly suppressed neuroinflammation in Parkinson’s disease model through miR-124-3p-FSTL1-NF-κB axis. Wang et al. (2021) showed that miR-124-3p significantly aggravated inflammatory reactions by promoting the activation of the TLR4/NF-κB signaling pathway in osteoarthritis. However, it remains unknown whether HOXA11-AS is involved in the progression of TBI by modulating the miR-124-3p-mediated TLR4/NF-κB pathway. A recent *in vitro* and *in vivo* study showed that the expressions of HOXA11-AS, MDK, TLR4, and NF-κB were significantly increased, while the expression of miR-124-3p decreased in the injured cortex of TBI rats (Li X. L. et al., 2022). Furthermore, HOXA11-AS overexpression aggravated neurological deficits by increasing brain edema and apoptosis by promoting the secretion of proinflammatory factors, including interleukin-1β and interleukin-6 in TBI rats (Li X. L. et al., 2022). Additionally, miR-124-3p overexpression or MDK downregulation repressed the inflammatory response of astrocytes (Li X. L. et al., 2022). Importantly, the overexpression of HOXA11-AS inhibited the expression of miR-124-3p and promoted the expression of MDK, TLR4, and NF-κB in TBI rats (Li X. L. et al., 2022). Interestingly, the anti-inflammatory effects of miR-124-3p were reversed by HOXA11-AS overexpression (Li X. L. et al., 2022). In contrast to the aforementioned observations, Yang et al. (2019) reported that EXO-miR-124 improved hippocampal neurogenesis by promoting the M2 polarization of microglia through the inhibition of the TLR4 pathway after TBI. The results of this study suggested that HOXA11-AS might play a role in promoting the inflammatory response of TBI by activating the MDK/TLR4/NF-κB signaling pathway through the inhibition of miR-124-3p. However, the non-conformity of the relationship between miR-124 and TLR4 requires further research.

#### miR-124 overexpression inhibits the development of TBI by suppressing inflammatory cytokine release through targeting VAMP-3

Cognitive dysfunction is a common neurological manifestation of TBI (Bray et al., 2022). The inflammatory cytokine release in the brain is associated with cognitive dysfunction. Xie X. et al. (2021) reported that Dexmedetomidine (Dex) significantly improved cognitive dysfunction by inhibiting the release of surgery-induced pro-inflammatory cytokines. The vesicle-associated membrane protein 3 (VAMP-3) has been implicated in various disease conditions by inhibiting nearly all the cytokine release including IL-6, IL-1β, and TNFα (Meng et al., 2019; Zhu et al., 2020). For instance, VAMP-3 alleviated inflammatory joint damage in arthritis by suppressing the release of IL-6 and TNFα (Boddul et al., 2014). VAMP-family has been found to be associated with the pathological process of TBI (Fu et al., 2022). A recent study demonstrated that miR-124 could inhibit the growth ability in non-small cell lung cancer cells and patient-derived xenograft mouse models by regulating the expression of VAMP-3 (Petrek et al., 2021). However, it is unclear whether miR-124 alleviates POCD of TBI patients by inhibiting the release of the cytokines through targeting VAMP-3. In an *in vitro* and *in vivo* study, Chen et al. (2019) found that miR-124 expression was decreased and the expression of VAMP-3 was upregulated in BV2 microglial cells following LPS stimulation. In addition, miR-124 mimics or VAMP-3 knock-down significantly suppressed the expression of IL-6 and TNF-α (Chen et al., 2019). Furthermore, increased miR-124 expression dramatically decreased IL-6 and TNF-α release related to microglial activation by decreasing the expression of VAMP-3 (Chen et al., 2019). Additionally, these findings indicated that miR-124 might be involved in the inflammation of neuronal cells and the development of TBI. However, further research is needed to establish direct evidence for this assumption.

#### The overexpression of miR-124 inhibits apoptosis of neuronal cell death and improves motor and memory dysfunction by the inhibition of the DAPK1-NR2B axis in TBI mice

As is well known, TBI can induce axonal injury and neuronal cell death (Mi et al., 2021). A late study developed by Zhuang et al. (2023) demonstrated that bone marrow stromal cells-derived exosomes (BMSCs-Exos) protected from neurological damage in TBI via the miR-124-3p/p38 MAPK/GLT-1 axis. Death-associated protein kinase 1 (DAPK1), a calcium/calmodulin-dependent serine/threonine kinase, plays a crucial role in regulating neuronal cell death (Zhang et al., 2022). The lack of DAPK1 suppresses neuronal cell death, whereas overexpression of DAPK1 induces neuronal cell death. DAPK1 knockdown not only significantly inhibited neuronal cell death, but also effectively attenuated the development of neuropathology (Kim et al., 2021). Additionally, it was reported that DAPK1 induced neuronal cell death through the N-methyl-D-aspartate (NMDA) receptor (Shi et al., 2021). NR2B, a subunit of NMDA receptors, has been shown to maintain neuronal plasticity and normal cellular functions. Previous evidence indicated that the inhibition of NR2B phosphorylation significantly rescued TBI-induced neurological impairment (Xu et al., 2019). Moreover, DAPK1 can bind to the NMDA receptor NR2B C-terminal tail. Tu et al. (2010) reported that genetic deletion of DAPK1 protected neurons against cerebral ischemic insults by blocking injurious Ca (^2+^) influx by targeting NR2B. Furthermore, DAPK1 was proven to be a direct target of miR-124. Shi et al. (2021) demonstrated that miR-124 significantly alleviated ischemic stroke-induced neuronal death by inhibiting DAPK1. However, it is unclear whether miR-124 is involved in TBI by regulating the DAPK1-NR2B axis. A recent clinical and animal study indicated that the expression of miR-124 was significantly decreased in the perilesional cortex of TBI mice, whereas the expression levels of the DAPK1 and NR2B in plasma of TBI patients and the perilesional cortex of TBI mice (Shi et al., 2022). Furthermore, a high level of DAPK1 expression or a low miR-124 expression in the plasma of TBI patients was associated with unfavorable TBI outcomes (Shi et al., 2022). However, overexpression of miR-124 or knockdown DAPK1 in the TBI mice significantly rescued TBI-induced motor and memory dysfunction (Shi et al., 2022). Also, overexpression of miR-124 or knockdown DAPK1 reversed the expression of phosphorylated NR2B (Shi et al., 2022). Tat-NR2B, a transmembrane peptide, can specifically inhibit the binding of NR2B and DAPK1 (Shi et al., 2022). Shi et al. (2022) found that the TBI mice showed decreased lesion volume after Tat-NR2B injection. The p-NR2B/NR2B expression was significantly decreased in TBI mice after Tat-NR2B injection, whereas DAPK1 remained unchanged (Shi et al., 2022). The expression levels of cleaved caspase3 and the apoptosis level were significantly reduced in the perilesional cortex after the Tat-NR2B injection (Shi et al., 2022). These findings suggested that overexpression of miR-124 reduced apoptosis in the perilesional cortex and significantly improved motor and memory dysfunction in TBI mice by inhibiting the DAPK1-NR2B axis.

#### miR-124 suppresses TBI by activating JAK/STAT3 and inhibiting JNK signaling pathway

Growth arrest-specific transcript 5 (GAS5), a long non-coding gene, has been found to be negatively associated with the expression of miR-124. Besides, lncRNA GAS5 was also found to be involved in the process of cerebral ischemia injury by regulating inflammation-related factors (Li et al., 2023). Long non-coding RNAs (lncRNAs) have multiple functions (e.g., mediating epigenetic changes, posttranscriptional regulation, and transcriptional regulation), and are proven to regulate many diseases (Xie W. et al., 2021; Gao et al., 2022). GAS5 is confirmed to regulate cell apoptosis, cell survival, and metabolic activities that participated in various diseases such as autoimmune diseases, cancers, and TBI (Lei et al., 2022; Peng and Huang, 2022; Wang H. et al., 2022). In addition, some miRNAs could be regulated by GAS5, such as miR-196a, miR-205, and miR-124 (Filippova et al., 2021; Li M. et al., 2022). Severe TBI not only damages the cerebrum but also leads to complex neurological impairments (Dever et al., 2022). Wang et al. (2018) reported that miR-124 overexpression significantly attenuated the invasion of renal cells by down-regulating STAT3. Sun et al. (2020) demonstrated that miR-124-3p suppressed the proliferation and motility of papillary thyroid cancer cells by inactivating the MAP2K4/JNK pathway. Therefore, miR-124 overexpression might alleviate TBI via the activation of JAK/STAT3 and the inhibition of the JNK signaling pathway. However, other studies demonstrated that miR-124-3p was downregulated and STAT3 expression was upregulated post-TBI (Vuokila et al., 2018, 2020a). Importantly, STAT3 upregulation correlated with the miR-124-3p downregulation (Vuokila et al., 2018, 2020a). Additionally, of the 30 miR-124-3p predicted targets, 9 fell within the STAT3 network (Vuokila et al., 2018, 2020a). Thus, the relationship between miR-124 and STAT3 requires further study in TBI.

#### Downregulation of miR-124 promotes SVZ NSC activation by enhancing the function of PI3K/AKT and Ras/MEK/Erk signaling pathways after TBI

The phosphatidylinositol 3-kinase/protein kinase B (PI3K/AKT) pathway is widely involved in the regulation of multiple diseases (Hu et al., 2021). PI3Ks belong to the family of intracellular lipid kinases and are also identified as upstream key elements involved in the response to the PI3K/AKT signaling pathway (Liu R. et al., 2020). PI3Ks are categorized into three classes (I-III), among which class I isoforms were most generally studied (Setiabakti et al., 2022). Protein kinase B (PKB), a serine/threonine kinase, is the core of the PI3K/AKT signal pathway (Xue et al., 2021). Different genes encode three protein isoforms of AKT (AKT1/PKBα, AKT2/PKBβ, and AKT3/PKBγ) (Basu and Lambring, 2021). The PI3K/AKT signaling cascade has been proven to play a crucial role in the central nervous system (Matsuda et al., 2019). During brain development, the PI3K/Akt signaling pathway is a key regulator of neuronal cell proliferation and dendritic formation (Kalra et al., 2022). PI3K/Akt signaling pathway has been shown to be involved in TBI. As reported, the PI3K/Akt signaling pathway is activated after TBI and therefore exerts neuro-protective effects by promoting neuronal survival and inhibiting apoptosis (Feng et al., 2022). Activation of Akt can regulate a variety of apoptosis-related proteins and pathways, such as inhibition of caspase family protein activation, to reduce neuronal apoptosis during TBI. He et al. (2018) reported that sevoflurane post-conditioning significantly attenuated TBI-induced neuronal apoptosis by modulating autophagy through the activation of the PI3K/AKT signaling pathway. In addition, several brain functions are modulated through renin-angiotensin system (Ras) receptors (Mirzahosseini et al., 2021). Also, Ras/MEK/Erk has been implicated in the development of TBI (Mirzahosseini et al., 2021). However, whether miR-124 is involved in the pathological development of TBI by regulating PI3K/AKT or Ras/MEK/Erk signaling pathway is unknown. Kang et al. (2022) assessed the expression of miR-124-3p in the subventricular zone (SVZ) in rats on days 1, 3, 5, 7, 14, and 30 post-TBI and showed a downregulation of miR-124-3p. Further study found that downregulation of miR-124-3p promoted neural stem cells (NSCs) activation in the SVZ after TBI and significantly improved motor function in adult rats with TBI (Kang et al., 2022). However, miR-124-3p agonists showed the reverse results (Kang et al., 2022). Moreover, the expression of Ras, MEK, Erk, PI3K, and Akt increased after TBI (Kang et al., 2022). More importantly, the expression of the aforementioned five proteins was significantly suppressed by the miR-124-3p inhibitor, while the miR-124-3p agonist promoted their expression (Kang et al., 2022). Therefore, the downregulation of miR-124-3p enhanced SVZ NSC activation and improved motor function through the activation of PI3K/AKT and Ras/MEK/Erk signaling pathways after TBI. Contrastingly, other investigators have shown the protective effect of miR-124 on TBI (Johnson et al., 2017; Yip et al., 2019; O’Connell et al., 2020).

## Discussion of the existing evidence

In this review, we have evaluated the existing evidence of mir-124 involvement in TBI, highlighting the different models that have been used to assess this, as well as the contradictory findings that have been reported. According to mounting clinical findings, various miRNAs have been found to be abnormally expressed in patients with TBI, such as miR-21, miR-137, miR-133, miR-199, miR-204, and miR-519 (Huang et al., 2023; Lin et al., 2023). Among the sixteen included studies, three clinical trials (O’Connell et al., 2020; Vuokila et al., 2020b; Shi et al., 2022) provided the clinical significance of miR-124 in TBI. Two of them (Vuokila et al., 2020b; Shi et al., 2022) reported that the expression of miR-214 was significantly lower in patients with TBI than the controls. Shi et al. (2022) demonstrated that miR-124 declined in the plasma of TBI patients with unfavorable outcomes compared to patients with favorable outcomes. Similar to Shi et al.’s (2022) finding, Vuokila et al. (2020b) showed that miR-124 was downregulated in the cortex of TBI patients by using *in situ* hybridization. However, inconsistent with the results of the above two studies, O’Connell et al. (2020) observed that the expression level of plasma miR-124-3p was elevated in patients with TBI via a bioinformatic analysis. In this preliminary study, miR-124 was one of the top six miRNAs identified to promise as blood biomarkers of TBI. The opposite results of plasma miR-124 expression in TBI patients between Shi et al. (2022) and O’Connell et al.’s (2020) study might be associated with distinct demographic characteristics (i.e., sample size, race, and regions), different disease states (i.e., early or late TBI, without specifically stated), and different experimental conditions assessing the miR-124 expression (i.e., measurements).

In addition, we could also find contradictory evidence for the molecular mechanisms of miR-124 in TBI among different studies. Since miR-124 is one of the early detected miRNAs in the family of non-coding RNAs, the biological functions of miR-124 are diversified (Lim et al., 2005). Thus, miR-124 may act on different target proteins via multiple mechanisms and regulate various signaling pathways. According to the current evidence, miR-124 plays an important role in central nervous system development, neuronal differentiation, neuroprotection, and tumor development. The most studied signaling cascades mediated by miR-124 included Wnt/β-catenin, Notch, MAPK/ERK, and PI3K/Akt. Therefore, the mentioned proteins and signaling pathways associated with mir-124 in this review were diverse, and so we can speculate that mir-124 plays an essential role in the development of TBI, but underlying molecular mechanisms are various and need further investigation.

## Conclusions and prospects

**FIGURE 1:**
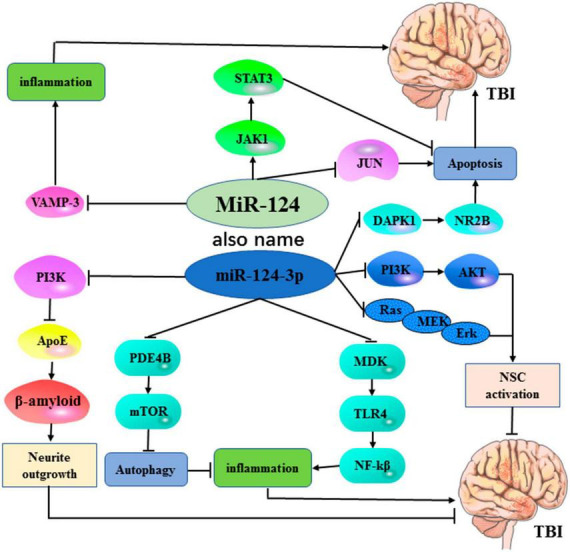
The molecular mechanisms underlying the essential roles of miR-124 in TBI.

Based on this review, miR-124 functions as a regulator involved in apoptotic cell death and cell proliferation and is also strongly associated with the pathophysiological development of TBI. Based on the studies included in this review, the downregulation of miR-124-3p might contribute to TBI-induced neurodegeneration and inflammation, whereas high expression of miR-124 might improve or mitigate the neuron injury of TBI. At present, some interventions have been found to elevate the level of miR-124, these include but are not limited to miR-124 inhibitors, microglial exosomes, electroacupuncture, docosahexaenoic acid, and brain-derived neurotrophic factor. The molecular mechanisms of the effects produced by miR-124 on TBI are illustrated in Figure 1. As shown in Table 1, only three out of sixteen (3/16, 19%) included studies reported the clinical applications of miR-124 in TBI, which limited its broad prospects. As a potential biomarker, early laboratory testing on the expression of miR-124 in patients with TBI may take that management a step further and apply it to judge the prognosis of the sufferers. In the way of treatment, currently, a miRNA nanocarrier system has already been developed, which supports the clinical feasibility of such miR-124-based therapy for treating TBI. Despite its potential for therapeutic application, the effect of miR-124 on TBI appears to be paradoxical. Following TBI, miR-124-3p inhibits motor function by suppressing the PI3K/AKT and Ras/MEK/Erk signaling pathways after TBI. miR-124-3p also exerts a protective role in TBI through modulating multiple signaling pathways, such as PDE4B/mTOR, JAK/STAT3, and DAPK1-NR2B signaling pathways.

Since miR-124 plays a pivotal role in TBI development by regulating multiple signaling cascades, specific drugs or biological compounds targeted by miR-124 and its pathways may effectively improve the impairment caused by TBI. During TBI, the release of multiple cytokines, neurotransmitters, and inflammatory mediators leads to the abnormal activation of a series of signaling pathways, these included, but were not limited to the Wnt/β-catenin, Notch, MAPK/ERK, and PI3K/Akt. These signaling cascades might be involved in inflammatory response, apoptosis, cell survival, and other important biological processes. Therefore, inhibitors or agonists designed for targeting these signaling pathways may recover the process of TBI by exerting the biological effects of anti-inflammation, neuroprotection, and angiogenesis promotion. However, it is important to acknowledge that the effectiveness of these inhibitors or agonists targeting signaling pathways in TBI primarily relies on evidence derived from laboratory studies and animal models. Therefore, some challenges remain in the clinical application of these inhibitors.

Although the preliminary understanding of the impact of miR-124 on TBI has been established, further investigation is required to fully comprehend the specific mechanisms underlying miR-124’s role in TBI. Additionally, comprehensive studies on a larger scale are necessary to evaluate the clinical significance of miR-124 as a potential therapeutic target for TBI. We posit that prospective clinical trials will play a crucial role in facilitating the translation of these findings into clinical practice for the treatment of TBI.

## Author contributions

PW: Investigation, Software, Writing – original draft. BH: Formal analysis, Supervision, Writing – review and editing. XL: Conceptualization, Writing – original draft, Writing – review and editing. HZ: Data curation, Writing – original draft, Writing – review and editing.

## Footnotes

1
www.mirbase.org

## Supplementary material

Supplementary Figure 1 Flow chart of study selection.
