# Supplementary figures and images for: Roles of microRNA-124 in traumatic brain injury: a comprehensive review
Source: Front Cell Neurosci. 2023 Nov 28;17:1298508. doi: 10.3389/fncel.2023.1298508 (PMC10687822; doi:10.3389/fncel.2023.1298508)

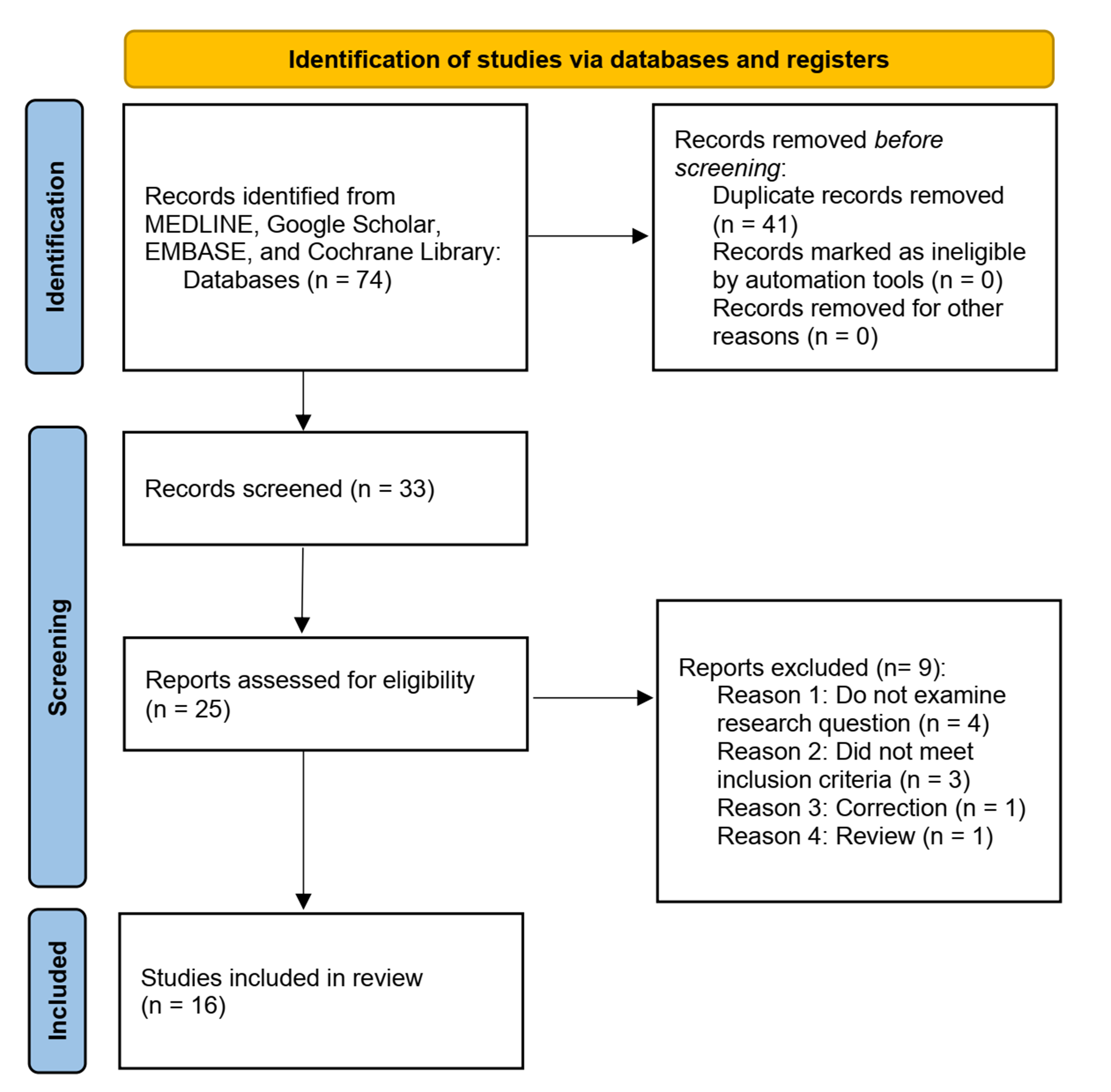

Supplement: Supplementary Figure 1 — Flow chart of study selection. [file Image_1.TIF]
